# Methodologies for the Emulation of Biomarker-Guided Trials Using Observational Data: A Systematic Review

**DOI:** 10.3390/jpm15050195

**Published:** 2025-05-10

**Authors:** Faye D. Baldwin, Rukun K. S. Khalaf, Ruwanthi Kolamunnage-Dona, Andrea L. Jorgensen

**Affiliations:** 1 Department of Health Data Science, University of Liverpool, Liverpool L69 3GL, UK; kdrr@liverpool.ac.uk (R.K.-D.); aljorgen@liverpool.ac.uk (A.L.J.); 2 Department of Public Health, Policy and Systems, University of Liverpool, Liverpool L69 3GL, UK

**Keywords:** biomarker-guided trials, target trial emulation, personalised medicine

## Abstract

**Background**: Target trial emulation involves the application of design principles from randomised controlled trials (RCTs) to observational data, and is particularly useful in situations where an RCT would be unfeasible. Biomarker-guided trials, which incorporate biomarkers within their design to either guide treatment and/or determine eligibility, are often unfeasible in practice due to sample size requirements or ethical concerns. Here, we undertake a systematic review of methodologies used in target trial emulations, comparing treatment effectiveness, critically appraising them, and considering their applicability to the emulation of biomarker-guided trials. **Methods**: A comprehensive search strategy was developed to identify studies reporting on methods for target trial emulation comparing the effectiveness of treatments using observational data, and applied to the following bibliographic databases: PubMed, Scopus, Web of Science, and Ovid MEDLINE. A narrative description of methods identified in the review was undertaken alongside a critique of their relative strengths and limitations. **Results**: We identified a total of 59 papers: 47 emulating a target trial (‘application’ studies), and 12 detailing methods to emulate a target trial (‘methods’ studies). A total of 25 papers were identified as emulating a biomarker-guided trial (42%). While all papers reported methods to adjust for baseline confounding, 40% of application papers did not specify methods to adjust for time-varying confounding. **Conclusions**: This systematic review has identified a range of methods used to control for baseline, time-varying, and residual/unmeasured confounding within target trial emulation and provides a guide for researchers interested in emulation of biomarker-guided trials.

## 1. Introduction

While randomised controlled trials (RCTs) are considered the gold standard for identifying causal relationships, in some scenarios they may not be a feasible, ethical, or cost-effective option [1]. In circumstances where it is not possible to carry out an RCT, observational studies may be used to examine the effectiveness of an intervention. However, due to a lack of randomisation, observational studies are prone to confounding bias, making the ability to infer causal relationships from such research challenging [2]. Furthermore, immortal time bias, which results from the failure to align the start of follow-up with the time that eligibility criteria are met and treatment is assigned, is another common source of bias that affects the validity of observational research [3]. The target trial emulation framework has been proposed to address these challenges, and involves specifying the hypothetical trial that would be conducted to investigate treatment effectiveness (the “target trial”) and comparing this with the observational data proposed to emulate the target trial, allowing researchers to identify potential sources of bias at the design stage [1]. Many different methodologies aiming to control for different sources of bias within an emulated trial have been proposed, and it can be challenging for researchers to identify the most appropriate methods to use, particularly as there are no systematic reviews that critically review and compare them.

Biomarkers, characteristics that are measured and evaluated as an indicator of a biological process, a pathogenic process or a pharmacological response, are increasingly used within research aimed at tailoring treatment to an individual—an area of personalised medicine. This growing area of research often adopts biomarker-guided trials—trials that incorporate one or more biomarkers in their design to either guide treatment and/or determine eligibility—to demonstrate the utility of using the biomarker(s) to inform treatment [4]. However, many biomarker-guided trial designs are unfeasible in practice, for example where long-term outcomes are studied, or when the outcome and/or biomarker is particularly rare, thus requiring unachievable sample sizes. Biomarker-guided trials often have complex designs involving large numbers of treatment arms and subgroups, which can result in insufficient power to detect a clinically meaningful difference [5,6,7]. Challenges associated with conducting biomarker-guided trials, including the cost and complexity of biomarker analysis, difficulty in estimation of recruitment rates, and dropout of participants with advanced disease, have been reported within the literature [8]. Additionally, the incorporation of dynamic treatment strategies, where treatment decisions change over time based on an individual’s biomarker status or treatment response, can be difficult to implement within biomarker-guided trials due to logistical and analytical challenges. This is particularly the case when such strategies rely on biomarker thresholds that are not known a priori, either requiring estimation during the trial and/or the requirement of additional treatment arms, whereby participants are randomised to different biomarker thresholds or biomarker-guided treatment strategies. These complexities may also raise ethical concerns, especially when preliminary evidence suggests potential benefit or harm in a biomarker-defined subgroup but lacks definitive validation.

Echoing the reasons outlined above, emulation of a biomarker-guided target trial using observational data could be a viable alternative in situations where it is not feasible to run a biomarker-guided clinical trial. Emulation of biomarker-guided trials may be particularly suited to the study of dynamic treatment strategies that would be unethical to implement within a clinical trial, including the effect of initiating, stopping, or switching treatment based on biomarker response. Emulation of biomarker-guided trials that compare dynamic treatment regimens could help address clinical questions that are unfeasible or unethical to study in randomised trials. This approach is particularly applicable to critical care settings, where randomisation is not feasible or ethical, and rapid clinical decisions are required. For example, emulation of a biomarker-guided trial could be used to determine the best time to initiate antibiotic therapy based on procalcitonin-guided strategies in patients with sepsis, incorporating the study of rapid implementation of treatment alongside the minimisation of unnecessary antibiotic use [9].

The objective of this systematic review was to identify and evaluate the various methodologies that have been proposed and/or used in target trial emulation studies comparing treatment effectiveness, with a focus on methods to control for bias from baseline, and time-varying, unmeasured, and residual confounding, in addition to immortal time bias. Furthermore, we identified emulated target trials utilising biomarkers and considered the applicability of the methods used to control for bias within emulated target trials more generally to those featuring biomarkers.

## 2. Materials and Methods

The Preferred Reporting Items for Systematic Reviews and Meta-Analyses (PRISMA) statement and checklist were followed within this manuscript (Supplementary Table S2). A protocol was written before starting the review and registered on the Open Science Framework (OSF) after the review was completed on 12 March 2025 (registration number: 92vhq) [10].

### 2.1. Eligibility Criteria

We included studies published in English which either detail the emulation of a target trial of treatment effectiveness using observational data (e.g., prospective and retrospective cohort studies, case-control research studies, real-world data registries, or databases), classified as ‘application studies’, or studies which propose methodology to be used within target trial emulation using observational data, classified as ‘methods studies’. Editorials, letters, commentaries, reviews, conference abstracts, case reports, and papers that did not detail the methods used to emulate a target trial were excluded, as well as studies not using observational data to emulate a target trial, for example those which used data exclusively from previous randomised controlled trials. Studies not assessing treatment effectiveness were also excluded. Additionally, animal studies were excluded.

### 2.2. Search Strategy

Four bibliographic databases (PubMed, Scopus, Web of Science, and Ovid MEDLINE) were searched for articles published up to 17 March 2023, using predefined search terms relating to target trial emulation and observational data (Supplementary File S1).

### 2.3. Data Collection

Search results were entered into EndNote X9 reference management software for the removal of duplicates before being exported and screened in two stages using Rayyan [11]. Two reviewers (FDB and RKS) independently undertook screening of titles, abstracts and full-text articles against the inclusion criteria. In the event of uncertainty regarding eligibility, discrepancies were resolved through discussion between reviewers and referral to third and fourth reviewers (ALJ and RDK).

Due to time constraints, we randomly selected 50% of the application papers for inclusion in the review, along with all remaining methods papers. Application papers were chosen by inputting their record numbers into R version 4.3.1 and using the “sample()” function to select a random subset.

### 2.4. Data Extraction

A data extraction spreadsheet was created specifically to extract data from all eligible studies by two reviewers (FDB and RKS). Data was extracted regarding:

1. Basic information about the study;
2. Study design, including specifics of the trial design emulated;
3. Statistical analysis, including details of statistical tests applied;
4. Methods to control for confounding/biases, including specific details and rationale of methods to adjust for baseline, time-varying, unmeasured and residual confounding, alongside immortal time bias
5. Outcomes measured;
6. Use of biomarkers, including details of any biomarkers measured, and whether the study would be identified as a biomarker-guided trial, as defined above.

### 2.5. Data Analysis

A descriptive analysis of studies included in the review was undertaken, together with a narrative description of the methods detailed in the papers, in addition to a critique of their relative strengths and limitations.

## 3. Results

**Figure 1:**
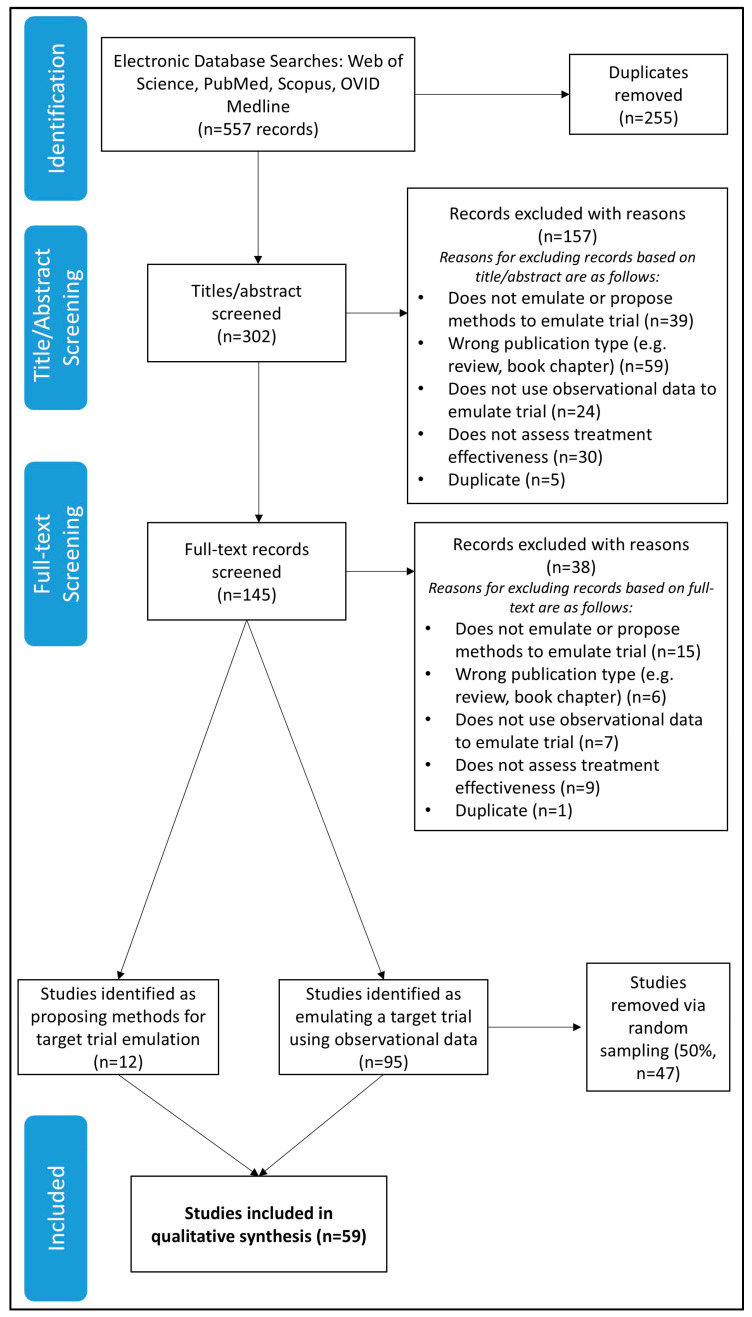
PRISMA flow chart.

The flow of studies in this review is presented in Figure 1. A total of 557 records were retrieved from all databases. After removing duplicates, 302 titles and abstracts were screened, and 157 were excluded; reasons for exclusion included not emulating or proposing methods for trial emulation (*n* = 39), not using observational data to emulate trial (*n* = 24), not assessing treatment effectiveness (*n* = 30), wrong publication type (*n* = 59), or being a duplicate record (*n* = 5). The full text of 145 papers were retrieved, with a further 38 excluded based on full-text screening; reasons for exclusion included not emulating or proposing methods to emulate trial (*n* = 15), not using observational data to emulate trial (*n* = 7), not assessing treatment effectiveness (*n* = 9), wrong publication type (*n* = 6), or being a duplicate record (*n* = 1). Due to time constraints, we randomly selected 50% of the application papers to include in the review, using the R function “sample()” while including all remaining methods papers. This resulted in 47 application studies [12,13,14,15,16,17,18,19,20,21,22,23,24,25,26,27,28,29,30,31,32,33,34,35,36,37,38,39,40,41,42,43,44,45,46,47,48,49,50,51,52,53,54,55,56,57,58] and 12 methods studies in the final review [59,60,61,62,63,64,65,66,67,68,69,70].

### 3.1. Observations from the Included Studies

#### 3.1.1. Specification of the Target Trial Protocol

Of the application studies, 29 (62%) referred to a target trial protocol, included commonly in the methods or supplementary sections, of which 4 (9%) protocols were published in clinical trial registries or research repositories. Of the methods papers, seven (58%) papers also specified a target trial protocol, again either in the methods or supplementary material sections, when specifying the target trial protocol.

#### 3.1.2. Specification of Causal Contrasts of Interest

Causal contrasts of interest are a key component of the target trial protocol, and are clearly defined comparisons made between different treatment strategies as they would ideally be implemented in a hypothetical RCT [1]. The most commonly assessed causal contrasts within RCTs involve the effect of being randomised to a treatment strategy, the intention-to-treat effect (ITT), and the effect of receiving the treatment strategy as specified in the protocol, the per-protocol effect, which excludes individuals who deviate from the protocol, drop out, or become non-adherent [1].

Of the application papers, the most assessed causal contrast of interest was a combination of the intention-to-treat and per-protocol effects (*n* = 14, 30%), followed by the per-protocol effect alone (*n* = 12, 26%) and the intention-to-treat (ITT) effect alone (*n* = 11, 23%). Other causal contrasts of interest included assessment of an as-treated approach (*n* = 1, 2%), the effect of the treatment a participant received, and assessment of an as-started approach (*n* = 2, 4%), the effect of the initially commenced treatment. Of the methods papers, the majority described assessment of the per-protocol effect (*n* = 5, 42%), followed by the intention-to-treat effect (*n* = 4, 33%). Several applications (*n* = 7, 15%) and methods papers (*n* = 2, 16%) did not specify the causal contrast of interest, an important component of the target trial protocol.

#### 3.1.3. Specification of Follow-Up

While most application papers specified the length of follow-up (*n* = 45, 96%), a lower number specified the time zero of follow-up (*n* = 36, 77%). All methodological papers specified the length of follow-up (*n* = 12, 100%), and most methods papers specified the time zero of follow-up (*n* = 11, 92%). We classified a paper as specifying time zero if it was clear that the start of follow-up aligned with meeting the eligibility criteria and being assigned to a treatment strategy (i.e., we did not require specific reporting of ‘time zero’ within the text).

### 3.2. Methods to Control for Confounding

#### 3.2.1. Baseline Confounding

Baseline confounding arises when one or more pre-intervention prognostic variables (a variable measured before starting the intervention of interest) are predictive of the intervention received at baseline and the start of follow-up [71]. Failure to adjust for baseline confounding can result in biased estimates of the association between exposure and outcome, and can result in invalid conclusions made about the identification of causal relationships. Baseline confounding cannot be directly solved by target trial emulation and requires the use of specific methods to appropriately adjust for it [72].

All application studies reported the use of at least one statistical method to control for baseline confounding, which was most achieved through the use of inverse probability weighting (IPW) within a marginal structural model (IPW-MSM, *n* = 19, 40%). Other methods to adjust for baseline confounding included standard adjustment within a regression model (*n* = 18, 38%), propensity score matching (*n* = 10, 21%), the clone-censor-weight method (*n* = 9, 19%), inverse probability of treatment weighting (IPTW, *n* = 7, 15%), and the parametric G-formula (*n* = 4, 9%). Most application studies used more than one method to control for baseline confounding (*n* = 22, 51%).

Similarly, all methods studies presented the use of at least one method to control for baseline confounding. This was also mostly achieved by use of IPW within a marginal structural model (*n* = 4, 33%) or the clone-censor-weight method (*n* = 4, 33%), followed by the parametric G-formula (*n* = 3, 25%). Half of methodological studies utilised more than one method to control for baseline confounding (*n* = 6, 50%). Details of the main methodologies applied to control for baseline confounding in both application and methods studies, alongside their advantages and disadvantages, can be found in Table 1.

#### 3.2.2. Time-Varying Confounding

Time-varying confounding occurs when a confounder changes over time and is associated with both the exposure (e.g., treatment) and the outcome. Examples of time-varying confounders include biomarker measurements, such as BMI and blood pressure. Time-varying confounding often arises alongside time-varying exposures, such as treatment dose [3]. In personalised medicine, time-varying confounding by prior exposure is a common challenge due to the dynamic nature of biomarker measurements. Prior biomarker levels often influence treatment decisions at baseline, and biomarker levels post-baseline can influence the nature of the exposure, such as treatment, and its relationship with the outcome [72]. Time-varying confounding cannot be solved by target trial emulation alone or conventional methods to control for confounding, and requires the use of more statistically advanced, generalised ‘G-methods’, which appropriately model the effect of time-varying confounders on outcome [3].

Most application studies reported using at least one method to control for time-varying confounding (*n* = 28, 60%). The most commonly used method was inverse probability weighting (IPW) within a marginal structural model (*n* = 19, 40%), followed by the clone-censor-weight method (*n* = 8, 17%), implementation of a sequential trial design (*n* = 8, 17%), inverse probability of censoring weighting (IPCW, *n* = 4, 9%) and the parametric G-formula (*n* = 4, 9%). Several studies used more than one method to control for time-varying confounding (*n* = 16, 34%). Over a third of studies (*n* = 18, 38%) did not report a method to control for time-varying confounding.

Similarly, the majority of methods studies presented the use of at least one method to control for time-varying confounding (*n* = 11, 92%). Methods included the use of IPW as part of an MSM (*n* = 4, 33%), the clone-censor-weight method (*n* = 4, 33%), IPCW (*n* = 3, 25%), and the parametric G-formula (*n* = 3, 25%). Most methods studies utilised more than one method to adjust for time-varying confounding (*n* = 7, 58%). Details of the main methodologies applied to control for time-varying confounding in both application and methods studies, alongside their advantages and disadvantages, can be found in Table 1.

#### 3.2.3. Residual and Unmeasured Confounding

Unmeasured confounding occurs when a variable related to both the exposure and the outcome is not measured or adjusted for in an analysis. Residual confounding refers to bias due to measurement error following adjustment. Neither type of confounding can be directly resolved by target trial emulation and therefore requires the adoption of methods to appropriately adjust for them.

Most application studies reported one or more methods to control for residual and/or unmeasured confounding (*n* = 42, 89%), the most common method being use of sensitivity analyses (*n* = 36, 77%), followed by use of negative outcome controls (*n* = 10), creation of directed acyclic graphs (DAGs) to identify unmeasured confounders (*n* = 7, 15%), and calculation of the E-value for unmeasured confounding (*n* = 6, 13%). Other methods included the use of tracer outcomes (*n* = 2, 4%), instrumental variable analysis (*n* = 1, 2%), and use of positive outcome controls (*n* = 1, 2%). A minority of application studies did not specify a method to control for residual and/or unmeasured confounding (*n* = 5, 11%).

Similarly, the majority of methods studies presented use of at least one method to control for residual and/or unmeasured confounding (*n* = 7, 58%), using sensitivity analyses (*n* = 5, 42%), DAGs (*n* = 5, 42%), or negative outcome controls (*n* = 1, 8%) to minimize risk of residual and/or unmeasured confounding. Likewise, a minority of studies did not report or describe the use of a method to control for residual and/or unmeasured confounding (*n* = 5, 42%).

#### 3.2.4. Use of Biomarker-Guided Trial Designs

Of the application studies, 31 reported measurements of a biomarker in their target trial (*n* = 31, 66%). Of these, 20 incorporated one or more biomarkers in their design (*n* = 20, 43%). Of the papers that incorporated biomarkers in their design, 7 (35%) used a biomarker to determine eligibility, 2 (10%) used a biomarker to guide treatment, and 11 (55%) used a biomarker both to determine trial eligibility and to guide treatment.

Of the methods studies, 6 reported and/or described measurements of a biomarker in a target trial (*n* = 6, 50%). Of these, five papers (83%) incorporated one or more biomarkers in their design. Of the papers that incorporated biomarkers in their design, two (40%) used a biomarker to determine eligibility, and three (60%) used a biomarker both to determine trial eligibility and to guide treatment. Characteristics of studies identified as biomarker-guided trials can be found in Supplementary Table S1.

## 4. Discussion

### 4.1. Overview

This systematic review identified, summarised, and critically appraised the methods used and/or described to emulate a target trial comparing the effectiveness of treatments using observational data. A total of 59 studies were included, of which 47 applied methods to emulate a target trial (‘application studies’) and 12 described and/or utilised methods to emulate a target trial (‘methods studies’). A range of statistical methods used to account for baseline and time-varying confounding, as well as immortal time bias within the included studies, were identified and are summarised in Table 1.

While all application studies emulated a target trial, over a third (38%) did not specify the target trial protocol, the main component of the target trial framework. Failure to specify the target trial protocol raises questions regarding the validity of the target trial emulation and what an ideal hypothetical trial would look like if conducted in a real-life scenario. Furthermore, a failure to specify the target trial protocol means that key components of the target trial framework, including specification of the time zero of follow-up and causal contrasts of interest, are not specified. Without a clear definition of the start of follow-up, it is challenging to determine whether treatment assignment and the initiation of follow-up align as they would in a true randomised trial [2,72]. Furthermore, failure to align treatment assignment with the start of follow-up can result in immortal time bias, whereby participants assigned to the treatment group have a period of follow-up during which they cannot experience the event of interest, where they are deemed ‘immortal’, which can result in biased treatment effects [2,72].

Some studies also failed to specify the causal contrasts of interest, such as whether the target trial estimates the intention-to-treat effect (the effect of treatment assignment regardless of adherence) or the per-protocol effect (the effect of adhering to the assigned treatment strategy). A failure to specify these key components of the target trial protocol reduces the validity of a target trial, as it raises the question of whether the trial is truly being emulated, and whether results are different to those that would be achieved in a standard observational study, given that the target trial protocol is not fully specified. Furthermore, it casts doubt on whether the results of the emulated trial are comparable to those from randomised trials, which would specify their trial protocols in detail.

### 4.2. Applicability of Methods in a Biomarker-Guided Target Trial Setting

Target trial emulation offers a unique opportunity to compare and validate the clinical utility of new and existing biomarkers for guiding treatment decisions, particularly in situations where a randomised trial is unethical or unfeasible. Many of the studies which included biomarker measurements do not explicitly mention biomarker-guided trials or personalised medicine, despite fitting into these categories. While many real-life biomarker-guided randomised trials have focused on time-fixed genetic biomarkers to guide treatment decisions, many of the identified biomarker-guided target trials have utilised time-varying, non-genetic biomarkers, such as CD4 cell count, estimated glomerular filtration rate (eGFR), and low-density lipoprotein cholesterol (LDL-C) [8,20,21,26,27,30,49]. This may be due to the fact that these measurements are routinely collected in electronic health records (EHRs), often used for target trial emulation, whereas genetic biomarkers require genotyping, which is not as commonly conducted within healthcare settings, and are usually unavailable via EHRs.

Of note, many of the biomarker-guided target trials identified compared the effect of dynamic treatment strategies, where treatment initiation, discontinuation, and switching are dependent on biomarker thresholds. For example, Cain et al. compared the effect of dynamic regimes in the context of initiation of combined antiretroviral therapy (cART) dependent on CD4 cell count, specified as follows: ‘Initiate treatment within m months after the recorded CD4 cell count first drops below *x* cells/mm^3^’, where *x* takes values from 200 to 500 in increments of 10, and m takes values of 0 or 3 [20]. Comparing these different strategies would be challenging in a randomised trial, but feasible within an emulated target trial scenario. Furthermore, target trial emulation could be useful when there is uncertainty surrounding the most optimal biomarker threshold to use within a trial, and allows for the comparison of several biomarker thresholds, which would be difficult to do in a randomised trial.

Several studies used biomarker thresholds to determine time of trial entry and to guide treatment decisions, such as McGrath et al., who required a platelet count of ≤30 × 10^9^/L after treatment initiation to enter the trial, and Fu et al., who compared strategies of ‘stopping RASi within 6 months and remaining off treatment after an eGFR decrease <30 mL/min per 1.73 m^2^’ versus ‘continuing RASi for the entire follow-up’ [30,43]. Again, these strategies are challenging to implement in a randomised trial and within observational studies due to ethical concerns related to early treatment cessation and switching. However, applying the target trial framework to study biomarker-guided strategies helps mitigate design-related biases—such as immortal time bias and selection bias—by aligning treatment assignment with the start of follow-up [1]. Given that a recent review found 57% of observational studies suffer from immortal time bias, using the target trial framework to emulate biomarker-guided trials, rather than relying on standard observational analyses, could improve the validity of results by more closely approximating a randomised trial using observational data [72,78].

A common theme amongst biomarker-guided target trials, specifically studies that used a biomarker to guide treatment, was the use of methods to control for time-varying confounding, particularly the use of inverse probability weighting (IPW), either within a clone-censor-weight (CCW) design or as part of a marginal structural model (IPW-MSM). Biomarkers are often time-varying in nature and share complex relationships with other confounding variables, alongside past exposure to treatment. Use of standard statistical methods, such as regression, and even more advanced methods, such as random-effects models, have been shown to be biased in the presence of time-varying confounding [3]. To accurately adjust for the time-varying effect of the biomarker and prevent blocking the effect of past exposure to treatment on the biomarker, the use of causal ‘G-methods’ is recommended [3]. G-methods include inverse probability weighting, parametric and non-parametric G-computation (e.g., parametric G-formula), and G-estimation [3,84].

Alongside their ability to adjust for baseline and time-varying confounding, G-methods can also be used to compare counterfactual outcomes, allowing the emulation of ‘what would have happened’ situations depending on treatment strategies [3]. G-methods can be used to compare counterfactual outcomes in specific risk groups, such as individuals with a genetic predisposition to a disease, by predicting what would happen under different treatment or exposure scenarios. For example, the scenarios ‘What if all individuals with a genetic mutation experienced poor treatment response?’ and ‘Would implementation of a genotype-informed treatment strategy versus standard care reduce the risk of adverse events?’ could be implemented using G-methods. As such, the use of G-methods within an emulated biomarker-guided trial could be beneficial for researchers aiming to evaluate personalised treatment strategies.

## 5. Recommendations and Future Directions

Although most application studies specified a target trial protocol, 38% did not. Without explicitly defining the target trial and detailing how observational data is used for emulation, studies may be more susceptible to confounding. Full specification of the target trial protocol within a paper’s methods or supplementary section is recommended to enhance replicability. Alternatively, researchers could register the target trial protocol on a data sharing repository or a trial registration site. Within the target trial protocol, we also recommend specification of the causal contrasts of interest, such as whether intention-to-treat (ITT) and/or per-protocol (PP) effects were estimated. As the goal of target trial emulation is to emulate the ideal, hypothetical RCT, specifying causal contrasts of interest enhances both the replicability and validity of the emulated trial. A reporting guideline for researchers emulating a target trial (TARGET) is in development, which seeks to improve and standardise the approaches adopted by researchers in the field [86]. We anticipate that adherence to the TARGET guideline will improve the reporting and replicability of target trial emulation studies.

**Figure 2:**
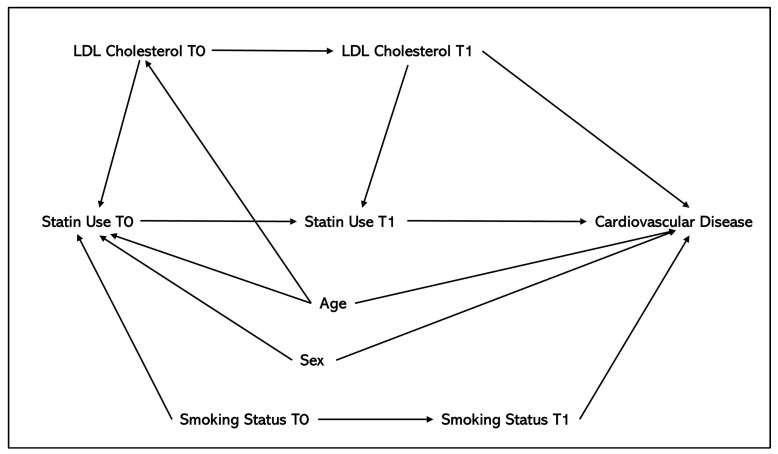
Directed acyclic graph (DAG) depicting the hypothesised causal relationships between statin use (exposure) and cardiovascular disease (outcome), accounting for time-varying statin use (at baseline, T0 and follow-up, T1) alongside time-varying variables LDL cholesterol and smoking status, and age and sex as baseline confounders.

A common limitation within application and methods studies was the presence of residual and unmeasured confounding. While many studies aimed to investigate the impact of residual and unmeasured confounding using sensitivity analyses, we recommend that researchers identify potential sources of confounding in the planning stages of target trial emulation through use of directed acyclic graphs (DAGs). DAGs, also known as causal graphs, present all assumed potential causal relationships between potential confounders, outcome, and the exposure/treatment, using unidirectional arrows [60,75]. DAGs can include measured and unmeasured confounders, alongside causes and effects of exposures and outcomes [60]. We particularly recommend the use of DAGs to illustrate potential confounders that change over time, such as biomarkers, and how these are affected by prior treatment, in addition to other confounders, and their effect on the outcome. An example of a simple DAG is shown in Figure 2. The DAG highlights the time-varying nature of statin use, measured both at baseline (T0) and follow-up (T1), and incorporates a time-varying biomarker, LDL cholesterol. The graph shows how baseline LDL cholesterol (LDL Cholesterol T0) influences statin use at baseline (Statin Use T0) and that follow-up LDL cholesterol (LDL Cholesterol T1) can be affected by prior statin use (Statin Use T0) via LDL cholesterol measurements at baseline. Furthermore, the DAG depicts the clinical feedback loop where LDL Cholesterol T1 directly influences Statin Use T1, representing a clinician’s decision to adjust statin treatment based on follow-up cholesterol levels. Additionally, the DAG accounts for smoking status as a time-varying confounder, measured at both baseline (Smoking Status T0) and follow-up (Smoking Status T1). Age is included as a covariate influencing baseline and follow-up LDL cholesterol, statin use, smoking status, and the development of cardiovascular disease, and sex is included as a covariate to account for potential sex differences in statin prescribing and cardiovascular disease risk. While this example DAG incorporates some key confounders, it does not represent unmeasured confounders (such as diet and physical activity) that could influence the relationships depicted.

We also recommend that researchers implement methods to identify residual and unmeasured confounding within their analysis. This could include investigation of negative controls (exposure controls and outcome controls), which may share the same sources of bias present in the original association between exposure and outcome, yet no causal effect is expected [1,87]. For example, in a target trial investigating the effect of antidepressants on severity of depression, a negative exposure control could include an alternative treatment which is not prescribed for depression, such as antihistamines, and a negative outcome control could include a health condition unrelated to depression, such as a broken bone [87]. In this example, the negative outcome control and exposure are not expected to influence depression severity, but may share common confounders with the exposure and outcome, such as age, gender or physical activity [87]. Alternatively, the E-value can be calculated to assess the level of unmeasured confounding within target trial emulation. The E-value assesses the minimal strength an unmeasured confounder would need to explain away an association, conditional on measured covariates [88].

We strongly recommend the use of advanced methods to account for time-varying confounding, particularly if a biomarker has been identified as a potential confounder, the biomarker is used as a measure of treatment response, and if aiming to emulate a biomarker-guided trial. If researchers are unsure of how to accurately adjust for time-varying confounders, several papers in the literature provide step-by-step instructions on methods for doing so, including clone-censor-weighting, sequential trial designs, marginal structural models, and the parametric G-formula [62,67,79,81,84,89]. Additionally, statistical packages designed for use in target trial emulation have been developed, including the R packages TrialEmulation, which combines data preparation for the emulation of sequential trials and calculation of inverse probability weights, and gfoRmula, which can be used to estimate the effects of sustained treatment strategies over time using the parametric G-formula [80,90].

While target trial emulation is a relatively new methodological framework, there are potential uses of target trial emulation that have not been identified in this systematic review. For example, future emulated trials could involve the use of other emerging methodologies such as machine learning, which could be used to predict treatment response dependent on biomarker information, such as physiological biomarkers (e.g., CD4 cell count, eGFR). Alternatively, use of artificial intelligence (AI) methods, such as AlphaFold3, a neural-network model used to predict the structure of biological molecules and their interactions, could be utilized alongside biomarker-guided trial emulation to better understand how treatments affect specific protein targets, or to help define biomarker subtypes based on protein structure and function (e.g., by identifying individuals with clinically relevant mutations) [91,92]. Emulated biomarker-guided trials could be used to assess the clinical utility of AI-based interventions, such as the clinical utility of AI in the interpretation of clinical images, such as X-rays, and could be extended to account for the time-varying nature of imaging data over the course of disease progression or treatment [93]. All of these applications of biomarker-guided trial emulation have potential for the ability to develop innovative personalised approaches to treating disease.

## 6. Conclusions

The implementation of the target trial emulation framework in observational research has enabled the estimation of causal effects that would otherwise be difficult or impossible to obtain, for example when conducting a trial would be unethical or unfeasible. Beyond standard two-arm trials, the use of target trial emulation has resulted in the emulation of trials with more complex designs, including comparison of static and dynamic treatment strategies dependent on levels of time-varying biomarkers, alongside trials with multiple treatment arms. However, to ensure that the results of target trial emulation would resemble those achieved in the ideal RCT, methods to adjust for baseline, time-varying, residual, and unmeasured confounding are required to be implemented while following the target trial framework. By doing so, emulated trials provide an efficient alternative to prospectively conducted trials and can greatly enhance the evidence base for both current and new treatments.

We believe that the target trial emulation framework provides unique opportunities for the demonstration of personalised approaches to treatments, in particular how biomarkers can be used to guide treatment strategies. We hope to see more biomarker-guided target trials in the future, especially in underfunded areas such as rare diseases.

## Figures and Tables

**Table 1:** Advantages and disadvantages of the methods used to control for confounding within application and methodological studies.

Method	Overview of Method	Types of Confounding Adjusted for	Number of Application Studies (N = 47)	References of Application Papers	Number of Methodological Studies (N = 12)	References of Methods Papers	Advantages	Disadvantages
Standard regression adjustment (adjusting for covariates in a regression model)	Inclusion of confounders as covariates in a regression model, e.g., logistic, linear, and Cox regression.	Baseline confounding only.	18	[13,16,17,18,19,20,23,25,26,27,31,32,33,37,38,41,42,45]	2	[60,67]	Easy to implement and interpret Less computationally intensive than other methods Versatile with other statistical approaches (e.g., machine learning)	Cannot adjust for time-varying covariates or their time-dependent relationship with both treatment and outcome as it assumes covariates remain fixed over time. Can introduce collider stratification bias when adjusting for a time-varying confounder that shares a common cause with the outcome [3]. Not appropriate when the exposure is time-varying, as it can introduce time-varying confounding by past exposure through over-adjustment bias [3].
Inverse probability of treatment weighting (IPTW)	Weighting individuals by the inverse probability of being assigned the treatment they were actually assigned to conditionally on the individual’s baseline covariates [73]. The weighted observations are then adjusted in a regression model (e.g., weighted logistic regression, weighted Cox model) for estimation of the average treatment effect (ATE).	Baseline and time-varying confounding	7	[15,40,44,45,48,53,56]	0	Not Applicable *	Mimics a randomised trial by creating a pseudo-population where treatment is independent of confounders [73]. Can be applied to time-varying exposures and confounders. Unlike propensity score matching, IPTW retains sample size by weighting observations instead of removing individuals who do not match [73]. Has been shown to reduce bias more effectively than standard regression adjustment [74].	Possibility of extreme weights if there are large differences in characteristics between groups, leading to biased results [73]. Requires extra adjustment for within-individual correlation and inflation of sample size (e.g., bootstrapping and robust variance estimation) [73]. Assumes there is no unmeasured confounding, that the propensity score model is correctly specified, and positivity (every individual must have some chance of receiving the treatment of interest) [75]. These assumptions may be difficult to understand for less-experienced researchers.
Propensity score matching (PSM)	Participants receiving the intervention are matched with those receiving the comparator based on similar propensity scores, which represent the probability of treatment assignment given observed covariates [76]. This approach estimates the Average Treatment Effect on the Treated (ATT). Covariate balance is assessed using the standardised mean difference (SMD).	Primarily baseline confounding; however, time-dependent propensity scores have been developed [77].	10	[29,32,33,34,35,36,39,49,56,57]	2	[59,70] **	Straightforward to implement and interpret. Less computationally intensive than other methods. Improves covariate balance without use of outcome data, allowing a separation of design from the analysis, which may be preferable to some researchers [76]. Can be applied to time-varying covariates using time-dependent PSM [77].	Removes unmatched individuals from analysis, reducing sample size. Can only improve balance on confounders provided they are included in the propensity score model [76]. Assumes there is no unmeasured confounding, that the propensity score model is correctly specified, and assumes positivity (every individual must have some chance of receiving the treatment of interest) [75]. These assumptions may be difficult to understand for less-experienced researchers.
Clone-censor-weight	Each individual is assigned to all treatment strategies compatible with their data at time zero, creating clones for each strategy. Clones deviating from their assigned strategy are artificially censored, and inverse probability of censoring weighting adjusts for the resulting selection bias [62].	Baseline, time-varying confounding and immortal-time bias.	9	[19,20,21,30,38,41,47,50,54]	4	[61,62,65,67]	Allows comparison of static and dynamic treatment strategies [62] Well documented within target trial emulation literature, more accessible than likes of other G-methods [62,67]	Can be computationally intensive if applied to large datasets. Assumes there is no unmeasured confounding, that the propensity score model is correctly specified, and assumes positivity (every individual must have some chance of receiving the treatment of interest). These assumptions may be difficult to understand for less-experienced researchers [78]. Requires extra adjustment for within-individual correlation and inflation of sample size (e.g., bootstrapping and robust variance estimation) [62].
Inverse probability of censoring weighting (IPCW)	To account for selection bias from artificial censoring, inverse probability of censoring weights are calculated for each individual at all time points, based on the probability of being uncensored, given prior exposure and censoring-related characteristics [62]. These weights are then used in the outcome model (e.g., weighted linear, logistic, or Cox regression).	Time-varying confounding only, but can be combined with IPTW in a marginal structural model or included as part of clone-censor-weighting to adjust for baseline and time-varying confounding.	10	[12,22,23,25,26,27,31,43,48,56]	3	[55,58,63]	Controls for bias introduced by censoring (loss to follow-up or dropout). Can be combined with other methods, including IPTW, PSM and standard covariate adjustment. Useful for modelling dynamic treatment strategies that include time-varying covariates or exposures.	Possibility of extreme weights if there are large differences in characteristics between groups, leading to biased results [73]. Assumes exchangeability, positivity, correct model specification and no unmeasured confounding. If these assumptions are not met, the model could fail to accurately adjust for selection bias [75].
Parametric G-Formula	The g-formula adjusts for time-varying confounders affected by prior exposures using a three-step algorithm. First, it models conditional probabilities from the observed data. Next, it uses these probabilities and baseline covariates to simulate time-varying covariates and outcomes via Monte Carlo sampling for each treatment group. Finally, the datasets are combined, and treatment effects are estimated by comparing hazard ratios across groups using a Cox model [79].	Baseline and time-varying confounding.	4	[17,21,47,58]	3	[64,66,68]	Flexibility to model complex treatment-confounder relationships across time, accounting for interactions between exposures and confounders, alongside modelling non-linear relationships [3,79]. Ideal for studies examining interventions on multiple risk factors (joint interventions) [3,79]. Dual role of adjusting for baseline and time-varying confounding. Provides estimates of counterfactual outcomes under different treatment scenarios and can be used to simulate real-world scenarios with complex, time-varying patterns of treatment use [79,80].	Harder to implement and interpret, may not be ideal for researchers less familiar with methods for time-varying confounding. Computationally intensive. Requires large sample sizes for more stable estimates, to minimise simulation error due to use of Monte Carlo sampling [79]. Requires explicit modelling of outcomes and covariates under specified treatment patterns, including whether they are static or dynamic, offering less flexibility than the likes of IPTW [3]. Reliant on correct model specification for outcome and confounder models [3,79].
Inverse probability weighting as part of a marginal structural model (IPW-MSM)	Rather than using a single treatment weight for baseline confounding, separate treatment weights are calculated at each time point, based on prior treatment, time-varying confounders, and baseline covariates, using a pooled logistic regression model [81]. A separate pooled logistic regression is used to calculate inverse probability of censoring weights for informative censoring. The final weights are obtained by multiplying treatment and censoring weights, which are then adjusted for in the outcome model (e.g., weighted logistic or Cox regression) [81].	Baseline, time-varying confounding and immortal time bias.	19	[14,20,21,22,25,26,27,30,31,36,38,42,46,47,48,51,52,53,58]	4	[61,64,65,68]	Allows estimation of unbiased estimates of treatment effects in longitudinal data, where confounders change over time because of treatment decisions [3]. Can be applied to both static and dynamic treatment regimes, making them ideal for comparing personalised treatment strategies or assessing the impact of interventions that depend on changing biomarkers or treatment decisions [3,62]. Provides robust estimates of treatment effects in longitudinal settings with time-varying treatments, making them useful for observational studies aiming to estimate the effect of treatment on long-term outcomes.	Can result in biased effect estimates if either outcome or weight models (e.g., propensity score models) are mis-specified [81]. Reliance on assumptions such as no unmeasured confounding, positivity (treatment must be possible for all individuals at all time points), and exchangeability (treatment assignment is independent of potential outcomes given confounders) [75]. Violations of these assumptions can compromise the validity of the results. Requires the calculation of inverse probability weights for each individual at each time point, which can be computationally demanding. Inter-individual correlation must be accounted for, as the same individual can contribute multiple observations. Additionally, to properly estimate the variability in treatment effects, methods such as bootstrapping and robust variance estimation are required [3,81].
Multiple imputation (MI)	Multiple imputation (MI) handles missing data by generating multiple datasets, where missing values are replaced with imputed values drawn from their predicted distribution based on available data [82].	Can be used to adjust for time-varying confounding by imputing missing confounder values, but is primarily used as a missing data technique.	1	[55]	1	[60]	Utilises all available data instead of discarding individuals with missing values, thereby maximising sample size and statistical power [82]. Reduces the risk of bias compared to complete case analysis, which removes individuals with missing data [82]. Compatible with various data types, including longitudinal, ordinal, and categorical data.	Does not explicitly adjust for time-varying confounding in the same way as g-methods (e.g., IPTW or the parametric g-formula), which are able to model relationships between treatment, confounders and outcome. As a result, use of MI alone may not fully account for time-dependent confounding, potentially leading to biased effect estimates. Assumes data is missing at random (MAR). If data is missing not at random (MNAR), results may be biased. This issue is particularly relevant when measuring biomarkers associated withadverse events, where missing data may be directly related to extreme or critical values [82]. Standard MI assumes independent observations. In the event of repeated measurements over time, an MI method that accounts for within-subject correlation should be used (e.g., standard fully conditional specification, joint multivariate normal imputation) [83]. Many MI methods assume normality, making imputation less reliable for skewed variables or extreme biomarker values; transformations (e.g. log transformation) are recommended [82,83]. Computationally intensive, especially when using large datasets or complex models.
Nonparametric Bayesian G-computation	Like the parametric G-formula, nonparametric Bayesian G-computation models conditional probabilities from observed data. Using Markov chain Monte Carlo sampling (MCMC), it simulates time-varying covariates and outcomes, with the flexibility to employ models such as Bayesian additive regression trees (BART) and Hilbert S pace Gaussian Processes to capture complex, high-dimensional relationships between covariates, outcome and treatment [69,84].	Baseline and time-varying confounding	1	[55]	1	[69]	Can model complex, non-linear relationships between covariates, outcomes and treatments without assuming a fixed functional form [69]. More flexible than the parametric G-formula, with no additional assumptions besides the no unmeasured confounding assumption [69]. Compatible with different types of outcome variables, including continuous, binary, categorical and time-to-event [84]. Allows prediction of counterfactual outcomes, allowing comparison of ‘what would have happened’ scenarios under different hypothetical interventions [69,84]. Applicable to joint interventions [84].	Computationally intensive due to requirement of MCMC and bootstrapping [84]. Results are harder to interpret compared to other methods such as standard regression, due to large number of possible hypothetical interventions compared [84]. Models may be incompatible with other parametric models, including MSMs, due to differences in model specification [84].
Exact matching	Matching treated individuals to controls with identical values for all covariates of interest [85].	Baseline confounding only	2	[32,33]	1	[63]	Simple to understand and implement. Less prone to bias from model misspecification [85]. Results are easier to interpret due to matched pairs being directly comparable.	Can lead to a significant reduction in sample size, as only individuals with exactly matching values across all covariates are retained [85]. Challenging to use in studies with many covariates or covariates with varying measurements, especially in biomarker-guided designs, where finding exact matches across biomarkers is difficult. Results in a large proportion of data being excluded when exact matches are not found. Low external validity, as exact matching on clinical factors may not be representative of real-world clinical practice.

* In the event of no studies (application or methods) reporting the method, the References column is defined as Not Applicable (NA). ** Time-dependent propensity score matching (time-dependent PSM).

## Data Availability

This systematic review did not generate or analyse any new datasets. The study synthesised findings from existing published literature, all of which are cited within the manuscript.

## Supplementary Materials

The following supporting information can be downloaded at: https://www.mdpi.com/article/10.3390/jpm15050195/s1, Table S1: Characteristics of biomarker-guided trials; File S1: Full search strategy used in each database; Table S2: PRISMA 2020 checklist.

## Abbreviations

The following abbreviations are used in this manuscript:

RCT	Randomised controlled trial
ITT	Intention-to-treat
PP	Per-protocol
IPW	Inverse probability weighting
IPTW	Inverse probability of treatment weighting
MSM	Marginal structural model
IPW-MSM	Inverse probability weighting as part of a marginal structural model
PSM	Propensity score matching
ATE	Average treatment effect
ATT	Average treatment effect on the treated
IPCW	Inverse probability of censoring weighting
SMD	Standardised mean difference
MI	Multiple imputation
MCMC	Markov chain Monte Carlo
BART	Bayesian additive regression trees
MAR	Missing at Random
MNAR	Missing Not at Random
EHR	Electronic health record
eGFR	Estimated glomerular filtration rate
DAG	Directed acyclic graph
TARGET	TrAnsparent ReportinG of observational studies Emulating a Target trial reporting guideline
AI	Artificial intelligence
