# Supplementary material for: Methodologies for the Emulation of Biomarker-Guided Trials Using Observational Data: A Systematic Review
Source: J Pers Med. 2025 May 10;15(5):195. doi: 10.3390/jpm15050195 (PMC12112824; doi:10.3390/jpm15050195)
Supplement: Supplementary file 1 [file jpm-15-00195-s001.zip › File S1 090525.pdf]

Database Search Strategy

1. **WEB OF SCIENCE**

((emulate OR emulated OR emulation OR emulating) AND (target_trial OR target_trials OR hypothetical_trial OR hypothetical_trials) AND observational)

Filter: 9.92 Statistical Methods (Citation Topics Meso)

Filter: English, Humans (via database interface)

2. **OVID MEDLINE**

((emulate OR emulated OR emulation OR emulating) AND (target trial OR target trials OR hypothetical trial OR hypothetical trials) AND observational.af.)

Limit to English language and humans

3. **SCOPUS**

(TITLE-ABS-KEY (emulate) OR TITLE-ABS-KEY (emulated) OR TITLE-ABS-KEY (emulation) OR TITLE-ABS-KEY (emulating))

AND

(TITLE-ABS-KEY (target_trial) OR TITLE-ABS-KEY (target_trials) OR TITLE-ABS-KEY (hypothetical_trial) OR TITLE-ABS-KEY (hypothetical_trials))

AND

(TITLE-ABS-KEY (observational))

AND

(LIMIT-TO (LANGUAGE, "English"))

Filter: Humans (via database interface)

4. **PUBMED**

((emulate OR emulated OR emulating OR emulation) AND (target trial OR target trials OR hypothetical trial OR hypothetical trials) AND observational)

Filter: English, Humans (via database interface)
