# Supplementary material for: Methodologies for the Emulation of Biomarker-Guided Trials Using Observational Data: A Systematic Review
Source: J Pers Med. 2025 May 10;15(5):195. doi: 10.3390/jpm15050195 (PMC12112824; doi:10.3390/jpm15050195)
Supplement: Supplementary file 1 [file jpm-15-00195-s001.zip › Supplementary Table S1 090525.pdf]

Supplementary Table S1. Characteristics of biomarker-guided trials

Author	Reference	Study type	Disease/Health condition	Treatment types compared	Treatment name(s)	Biomarker(s) measured	Role of biomarker(s)	How was biomarker used to guide	Methods to control for confounding	Dynamic treatment strategies?
Admon et al.	[59]	Methods	Hypoxemia	Endotracheal intubation (critical care medicine)	Positive-pressure ventilation with a bag-mask device (modified rapid sequence intubation, RSI) versus	SpO2 (oxygen saturation, %) BMI (Kg/m2), FiO2 (fraction of inspired oxygen, %)	Determine trial eligibility	Excluded individuals with SpO_2 <90% to distinguish preventive from rescue positive-pressure ventilation.	Propensity score matching (PSM), coarsened exact matching	No, static interventions compared (assignment to positive-pressure ventilation versus no ventilation).
Aubert et al.	[14]	Application	Hypertension	Antihypertensives	Angiotensin converting enzyme inhibitor (ACEI), beta blockers, calcium channel blockers	Systolic blood pressure (SBP, mmHg)	Determine trial eligibility	Included individuals with baseline SBP ≥130 mmHg to observe treatment intensification under new American Heart Association/American College of Cardiology (AHA/ACC) targets [14].	Inverse probability weighting within a marginal structural model (IPW-MSM)	No, static interventions compared (assignment to new medication versus maximising dose).
Boyne et al	[19]	Application	Pancreatic cancer	Chemotherapy (anti-cancer)	FOLFIRINOX, gemcitabine and nab-paclitaxel (GN)	Haemoglobin (g/L), platelet count (L), white blood cell count (g/L), serum creatinine (umol/L)	Determine trial eligibility	Patients were eligible if they met specific laboratory criteria at diagnosis or within 30 days, including haemoglobin > 80 g/L, platelet count > 100 × 10^9/L, white blood cell count > 4.0 g/L, and serum creatinine <100 µmol/L. If these measures were not met initially, patients could still participate once they achieved these levels within 8 weeks post-diagnosis.	Clone-censor-weighting	No, static interventions compared (initiation of FOLFIRINOX versus initiation of GN within 8 weeks of diagnosis).
Cain et al.	[20]	Application	Human Immunodeficiency Virus (HIV)	Antiretrovirals	Protease inhibitor (PI), nucleoside reverse transcriptase inhibitor (NRTI), boosted protease inhibitor (bPI), non-nucleoside reverse	HIV1-RNA (copies/mL), CD4 cell count (mm3)	Determine trial eligibility and guide treatment	Eligibility for the trial requires viral suppression (HIV-1 RNA ≤ 200 copies/ml within 360 days of treatment initiation), virological failure (two measurements of HIV-1 RNA > 200 copies/ml, 7-180 days apart), and a CD4 cell count within the last 90	Clone-censor-weighting, IPW-MSM	Yes, adjusts antiretroviral treatment based on HIV-1 RNA measurements across follow-up. If viral suppression fails or rises beyond certain thresholds (e.g., 400 copies/ml or 1000 copies/ml), treatment is switched.

					transcriptase inhibitor (NNRTI), fusion inhibitor (FI).			days. The study compares two treatment strategies guided by HIV1-RNA: the tight-control strategy, which involves treatment switching if HIV-1 RNA exceeds 400 copies/ml within 90 days, and the loose-control strategy, which switches at 1000 copies/ml.		
	[61]	Methods			No specific treatments specified			Individuals had to have HIV-RNA >500 copies/mL, CD4 cell count and HIV-RNA measurements within six months of each other, and CD4 cell count between 200 and 500 cells/mm3 with no history of CD4 cell count less than 500 cells/mm3 to enter trial. CD4 cell count was used as a biomarker of when to initiate treatment, based on outcomes of AIDS and death.		Yes, compared 31 dynamic treatment strategies of the form "initiate treatment within *m* months after the recorded CD4 cell count first drops below *x* cells/mm3", where *x* takes values from 200 to 500 in increments of 10 and *m* takes values 0 or 3.
Caniglia et al.	[21]	Application	Human Immunodeficiency Virus (HIV)	Antiretrovirals	Protease inhibitor (PI), nucleoside reverse transcriptase inhibitor (NRTI), boosted protease inhibitor (bPI), non-nucleoside reverse transcriptase inhibitor (NNRTI), fusion inhibitor (FI).	HIV1-RNA (copies/mL),CD4 cell count (mm3)	Determine trial eligibility and guide treatment	Individuals had to have confirmed virologic suppression (two consecutive HIV-RNA ≤ 200 copies/ml) within 12 months of initiating an eligible treatment regimen, and a CD4 cell count to enter trial. Used both CD4 cell count and HIV-RNA to guide treatment, comparing when monitoring frequency should be reduced (based on CD4 cell count) and when individuals should switch from first line to new	IPW-MSM, parametric G formula, clone-censor-weighting	Yes, compares 4 dynamic joint monitoring and treatment strategies:<br>1. CD4 threshold 350/tight control: monitor every 3-6 months when CD4< 350 cells/uL and every 9-12 months CD4 is above the threshold, switch within 3 months of HIV-RNA crossing above 200 copies/ml<br>2. CD4 threshold 350/loose control: same as (1) except HIV-RNA threshold is 1000 copies/mL<br>3. CD4 threshold 500/tight control: same as (1) except that

								regimen (based on HIV-RNA).		the CD4 cell count threshold is 500 cells/µl<br>4. CD4 threshold 500/loose control: same as (3) except that the HIV-RNA threshold is 1000 copies/ml.
	[22]	Application	Dementia	Statins	Simvastatin, pravastatin, fluvastatin, atorvastatin, cerivastatin, rosuvastatin	Total cholesterol (mmol/L), systolic blood pressure (SBP, mmHg), APOE ε4 carrier status	Determine trial eligibility	A recent total cholesterol measurement (within previous 3 years), and BMI and SBP measurements required to enter trial.	IPW-MSM, implementation of sequential trials design	No, static treatment strategies compared (statin initiation at baseline versus no statin initiation).
	[23]	Application	Adverse birth outcomes (stillbirth, preterm delivery, very preterm delivery, small-for-gestational-age (SGA) and very SGA,	Antiretrovirals	Zidovudine (ZDV), lamivudine (3TC) and nevirapine (NVP) combination (ZDV/3TC/NVP), tenofovir (TDF), emtricitabine (FTC), and efavirenz (EFV) combination (TDF/FTC/EFV)	CD4 cell count (mm3)	Guide treatment	The study emulated two comparisons:<br>1. Historical comparison (2004–2015): Compared women who initiated TDF/FTC/EFV (2012–2015) vs. ZDV/3TC/NVP (2004–2011).<br>2. Contemporaneous comparison (2009–2013): Compared women starting either regimen during a period when both were prescribed.<br>CD4 thresholds guided ART initiation, evolving over time: ≤200 cells/mm³, then ≤250 in 2008, and ≤350 in 2012, changing treatment	Inverse probability of censoring weighting (IPCW)	No, static treatment strategies compared (TDF/FTC/EFV versus ZDV/3TC/NVP). Focuses on comparing changes in clinical guidelines over time rather than dynamic treatment strategies, where treatment is adjusted based on biomarkers of treatment response and/or disease progression.

								decisions across these periods.		
Dickerman et al.	[26]	Application	Cancer (total cancer, seven site-specific cancers: female breast, colorectal, haematological, melanoma, lung, prostate, urothelial)	Statins	All statins prescribed within UK	Low-density lipoprotein cholesterol (LDL-C, mmol/L), high-density lipoprotein cholesterol (HDL-C, mmol/L), alanine aminotransferase (ALT, IU/L)	Determine trial eligibility and guide treatment	LDL-C <5 mmol L–1 required to enter trial and no hepatic impairment defined as a clinical code for hepatic failure or ALT ≥120 IU/L).<br><br>LDL-C used as a biomarker to guide treatment decisions across follow-up – when LDL-C ≥5 mmol, patients and their physicians will decide whether to start, stop, or switch therapy.	IPW-MSM, implementation of a sequential trial design	Yes, dynamic treatment strategies are compared, and treatment decisions adjusted based on whether LDL cholesterol ≥5 mmol/L across follow-up. During follow-up, treatment decisions (start, stop, or switch) are made by patients and clinicians based on clinical judgment.
	[27]	Application	Colorectal cancer						IPW-MSM	
Fu et al.	[30]	Application	Advanced chronic kidney disease (CKD)	Renin-angiotensin system inhibitors (RASi)	Angiotensin-converting enzyme inhibitors (ACEi), angiotensin receptor blockers (ARBs)	Estimated glomerular filtration rate (eGFR, ml/min per 1.73 m2), albumin-to-creatinine ratio (ACR), serum potassium (mmol/L), systolic blood pressure (SBP, mmHg), diastolic blood pressure (DBP, mmHg)	Determine trial eligibility and guide treatment	eGFR <30 ml/min/1.73m2 required to enter trial.<br><br>eGFR was used as a biomarker to guide treatment, with two strategies compared: "stop RASi within 6 months and remain off treatment after eGFR decreases to <30 ml/min per 1.73 m²" versus "continue RASi throughout the follow-up." As a secondary objective, the study compared stopping versus continuing RASi at different eGFR thresholds in two cohorts: one with a first detected eGFR decrease between 20 and 30 ml/min per 1.73 m²(higher eGFR cohort), and another with a first detected eGFR <20 ml/min per 1.73 m²(lower eGFR cohort).	IPW-MSM, clone-censor-weighting	Yes, dynamic treatment strategies are compared, and changes to treatment are made based on eGFR levels across follow-up.

Kalia et al.	[64]	Methods	Type 2 diabetes (T2D)	Antidiabetics (glucose-lowering medications)	Sodium-glucose co-transporter 2 inhibitors (SGLT-2i), dipeptidyl peptidase-4 inhibitors (DPP-4i)	Haemoglobin A1c (HbA_{1c}, %),	Determine trial eligibility	Individuals had to have elevated HbA1c (>8.5%) to enter trial.	IPW as part of MSM, parametric G formula	No, static treatment strategies compared (initiation of SGLT-2i versus standard care, and initiation of DPP-4i versus standard care).
Kuehne et al.	[38]	Application	Ovarian cancer	Chemotherapy (anti-cancer)	Second-line chemotherapy (LOT2)	Cancer antigen 125 (CA-125)	Determine trial eligibility and guide treatment	Disease progression after standard LOT1 treatment, which was defined as the doubling value of CA-125, required to enter trial.<br><br>Compared between immediate, never-treated, and delayed treatment, where treatment not based on biomarker increase (i.e., 6 weeks after progression defined by biomarker increase) was considered delayed treatment, with CA-125 guiding the decision of when to initiate or delay treatment.	IPW-MSM, IPCW	Yes, compares dynamic treatment strategies (immediate, versus never treated, versus delayed treatment), to evaluate if and when second-line chemotherapy should be provided.
	[65]	Methods	Cardiovascular disease	Statins		European Society of Cardiology Systematic Coronary Risk Evaluation (ESC SCORE, patient's 10-year risk of undergoing a fatal cardiovascular event based on age, sex, total cholesterol, smoking status	Determine trial eligibility and guide treatment	Individuals included in study when first shown to have crossed ESC SCORE (individual 10-year risk of undergoing a fatal cardiovascular event based on age, sex, total cholesterol, smoking status and blood pressure, and the LDL-C level) equals or exceeds 1%.<br><br>Compares different ESC-SCORE thresholds (immediate treatment when risk score crosses 1%, 2%, 3%, 5% and no initiation) to	Clone-censor-weight, IPW-MSM	No, treatment initiation is based on predefined, static risk score thresholds that are not based on disease progression or treatment response.

						and blood pressure)		determine when to start statin treatment, based on risk of major adverse cardiovascular events (MACE).		
Kwee et al.	[39]	Application	Hepatocellular carcinoma (HCC)	Chemotherapy (anti-cancer)	Yttrium trans-arterial radioembolisation (TARE), Sorafenib	Creatinine (mg/dL), bilirubin (mg/dL), alpha-fetoprotein (ng/mL), international normalised ratio (INR)	Determine trial eligibility	Individuals must have total bilirubin < 2.0mg/dl, INR < 1.7, and creatinine < 2.0mg/dl to enter trial.	PSM	No, static treatment strategies compared (assignment to Yttrium trans-arterial radioembolisation (TARE) versus standard of care (sorafenib, chemotherapy).
Lodi et al.	[66]	Methods	Human Immunodeficiency Virus (HIV)	Antiretrovirals (ART)	Protease inhibitors (PI), non-nucleoside reverse transcriptase inhibitors (NNRTIs)	CD4 cell count (cells/mm^3), HIV-RNA (copies/mL)	Determine trial eligibility and guide treatment	Individuals had to have 2 CD4 counts of >500 cells/mm3 within 90 days of each other to enter trial, and baseline HIV-RNA measurement.<br><br>Used CD4 cell count to guide treatment, comparing the strategies 'immediate ART initiation within 1 month of treatment assignment' versus 'deferred ART initiation within 1 month of second CD4 count<350 cells/mm3 or AIDS'.	Parametric G formula	Yes, compares effect of immediate treatment initiation (static strategy) versus delayed treatment initiation, where treatment initiated only after a second CD4 cell count <350 cells/mm3 or AIDS (dynamic strategy).
McGrath et al.	[43]	Application	Immune thrombocytopenia (ITP)	Thrombopoietin receptor agonists	Romiplostim	Platelet count ($\times 10^9$/L)	Determine trial eligibility	Used a treatment-decision design, whereby individuals entered the cohort when they had a platelet measurement of ≤ 30 x 109/L (anchor date), to reflect when treatment decisions are made in the management of ITP, based	Standardised morbidity ratio weighting, IPCW	No, static treatment strategies compared (assignment to romiplostim versus standard of care).

								on ITP practice guidelines [43].		
Schroeder et al.	[46]	Application	Cardiovascular disease	Antidiabetics	Long-acting insulin, short-acting insulin	Haemoglobin A1c (HbA_{1c}, %), estimated glomerular filtration rate (eGFR, mL/min/1.73 m), low-density lipoprotein cholesterol (LDL-C, mg/dL), blood pressure (mmHg)	Determine trial eligibility	Individuals with a qualifying HbA_{1c} level (6.8% to 8.5%) while receiving long-acting insulin were included in trial.	IPW-MSM	No, static treatment strategies compared (assignment to long-acting insulin only versus long-acting insulin plus short-acting insulin).
Smith et al.	[47]	Application	Prostate cancer	Anti-cancer	Androgen deprivation therapy (ADT)	Prostate-specific antigen (PSA), prostate-specific antigen doubling time (PSADT)	Determine trial eligibility and guide treatment	Individuals had to have a PSA relapse to enter the trial, defined as one of following: a PSA rise above 0.2 ng/mL beyond post-treatment nadir if initial treatment was prostatectomy (with or without radiation); a PSA that did not fall lower than 0.2 ng/mL if treated with prostatectomy with salvage radiation; or three successive PSA rises at least 30 days apart if initial treatment was only radiation.<br><br>Used PSADT as a biomarker to guide treatment, comparing strategies of the form "Start androgen deprivation therapy the first time PSADT drops below x days," where the threshold x varies from 0 to	IPW-MSM, parametric G-formula, clone-censor-weighting	Yes, dynamic treatment strategies compared, based on PSA levels, to determine when treatment should be initiated.

								360 in increments of 10, resulting in 37 different treatment strategies.		
Talmor-Barkan et al.	[49]	Application	Atrial fibrillation (AF)	Direct oral anticoagulants (DOACs)	Apixaban, rivaroxaban, dabigatran	Alanine aminotransferase (ALT, U/L), estimated glomerular filtration rate (eGFR, ml/min/1.73 m^2), haemoglobin A_{1c} (HbA1c, %), alkaline phosphatase (ALP, IU/L), platelets (PLTs, mcL), low-density lipoprotein cholesterol (LDL-C, mg/dL), creatinine (umol/L), aspartate transaminase (AST, U/L),	Determine trial eligibility	Individuals excluded from cohort if eGFR =< 30 mL/min/1.73 m^2.	Propensity score matching (PSM)	No, static treatment strategies compared (initiation of apixaban versus rivaroxaban versus dabigatran).
Trevisan et al.	[50]	Application	Hyperkalaemia	Mineralocorticoid receptor antagonists (MRA)	Spironolactone, eplerenone	Estimated glomerular filtration rate (eGFR, mL/min/1.73 m^2), plasma potassium (mmol/L)	Determine trial eligibility and guide treatment	Individuals excluded from cohort if missing creatinine measurements to estimate eGFR or plasma potassium. Compared risk of adverse events in individuals stopping versus continuing MRA in individuals with hyperkalaemia (plasma potassium > 5 mmol/L).	Clone-censor-weighting	No, static treatment strategies compared (assignment to stopping MRA versus continuing MRA within 6 months of hyperkalaemia).
Xie et al.	[53]	Application	Diabetic kidney disease	Antidiabetics	Sodium–glucose cotransporter 2 inhibitors	Estimated glomerular filtration rate	Determine trial eligibility	Individuals excluded from cohort if eGFR <15 mL/min/1.73 m2, or no	IPW-MSM, inverse probability of	No, static treatment strategies compared (assignment to either

					(SGLT2i), glucagon-like peptide 1 receptor agonists (GLP-1), dipeptidyl peptidase 4 inhibitors (DPP-4), and sulfonylureas	(eGFR, mL/min/1.73 m^2), haemoglobin A_{1c} (HbA1c, mmol/mol), low-density lipoprotein cholesterol (LDL-C, mg/dL), systolic blood pressure (SBP, mmHg), diastolic blood pressure (DBP, mmHg),		measurement of outpatient eGFR, haemoglobin A1c (HbA1c), height, weight, blood pressure, and LDL within the year before time zero.	treatment weighting (IPTW)	SGLTi, GLP-1, DPP-4 or sulfonylureas).
Xu et al.	[54]	Application	Hyperkalaemia	Renin-angiotensin system inhibitors (RASi)	Angiotensin-converting enzyme inhibitors (ACEi), angiotensin receptor blockers (ARB)	Estimated glomerular filtration rate (eGFR, mL/min/1.73 m^2), plasma potassium (mmol/L), urinary albumin-to-creatinine ratio	Determine trial eligibility and guide treatment	Individuals with first hyperkalaemia event (plasma potassium > 5 mmol/L) after RASi initiation and no missing eGFR values included.<br><br>Modelled the decision to stop/continue RASi after a potassium >5.0 mmol/L (mild hyperkalaemia), based on clinical guidelines and previous research [54]. Compares effect of higher potassium threshold (> 5.5 mmol/L) on stopping/continuing treatment as a secondary objective.	Clone-censor-weight	No, static treatment strategies compared (stopping RASi versus continuing RASi).
Yarnell et al.	[55]	Application	Acute hypoxemic respiratory failure	Invasive ventilation		Inspired oxygen fraction (FiO2), saturation-to-inspired oxygen ratio (SF), heart rate, respiratory	Determine trial eligibility and guide treatment	Individuals had to have FiO2 of =>0.4 or more via non-rebreather mask, non-invasive positive pressure ventilation (NIV), or high-flow nasal cannula (HFNC)	Nonparametric Bayesian G-computation	Yes, dynamic treatment strategies compared, uses saturation-to-inspired oxygen ratio (SF) thresholds to determine when to initiate invasive ventilation.

						rate, peripheral oxygen saturation, partial pressure of carbon dioxide (pCO2), pH, systolic blood pressure (SBP, mmHg), lactate		within 24 h of ICU admission to enter trial, alongside a pCO2 => 60, and a pH =< 7.20.<br><br>Compared three thresholds for initiation of invasive ventilation, based on saturation-to-inspired oxygen ratio (SF) of < 110, < 98, and < 88, on risk of 28-day mortality.		
Zhang et al.	[58]	Application	Anaemia	Erythropoiesis-stimulating agents	Epoetin alfa (EPO)	Haematocrit (%)	Guide treatment	Compared the effect of three dynamic epoetin dosing strategies based on haematocrit level: low haematocrit (adjust EPO dose to maintain haematocrit 30-33%), mid haematocrit (adjust EPO dose to maintain haematocrit 33-36%), and high haematocrit (adjust EPO dose to maintain haematocrit at 36-39%).	Parametric G-formula, IPW-MSM	Yes, dynamic treatment strategies compared, used haematocrit as a biomarker to evaluate effect of EPO dosing on survival outcomes.

References

14. Aubert, C.E.; Sussman, J.B.; Hofer, T.P.; Cushman, W.C.; Ha, J.K.; Min, L. Adding a New Medication Versus Maximizing Dose to Intensify Hypertension Treatment in Older Adults : A Retrospective Observational Study. *Ann. Intern. Med.* **2021**, *174*, 1666–1673.
19. Boyne, D.J.; Brenner, D.R.; Gupta, A.; Mackay, E.; Arora, P.; Wasiak, R.; Cheung, W.Y.; Hernán, M.A. Head-to-head comparison of FOLFIRINOX versus gemcitabine plus nab-paclitaxel in advanced pancreatic cancer: A target trial emulation using real-world data. *Ann. Epidemiol.* **2023**, *78*, 28–34.
20. Cain, L.E.; Saag, M.S.; Petersen, M.; May, M.T.; Ingle, S.M.; Logan, R.; Robins, J.M.; Abgrall, S.; Shepherd, B.E.; Deeks, S.G.; et al. Using observational data to emulate a randomized trial of dynamic treatment-switching strategies: An application to antiretroviral therapy. *Int. J. Epidemiol.* **2016**, *45*, 2038–2049.
21. Caniglia, E.C.; Robins, J.M.; Cain, L.E.; Sabin, C.; Logan, R.; Abgrall, S.; Mugavero, M.J.; Hernández-Díaz, S.; Meyer, L.; Seng, R.; et al. Emulating a trial of joint dynamic strategies: An application to monitoring and treatment of HIV-positive individuals. *Stat. Med.* **2019**, *38*, 2428–2446.
22. Caniglia, E.C.; Rojas-Saunero, L.P.; Hilal, S.; Licher, S.; Logan, R.; Stricker, B.; Ikram, M.A.; Swanson, S.A.; Emulating a target trial of statin use and risk of dementia using cohort data. *Neurology* **2020**, *95*, e1322–e1332.
23. Caniglia, E.C.; Zash, R.; Jacobson, D.L.; Diseko, M.; Mayondi, G.; Lockman, S.; Chen, J.Y.; Mmalane, M.; Makhema, J.; Hernán, M.A.; et al. Emulating a target trial of antiretroviral therapy regimens started before conception and risk of adverse birth outcomes. *Aids* **2018**, *32*, 113–120.
26. Dickerman, B.A.; García-Albéniz, X.; Logan, R.W.; Denaxas, S.; Hernán, M.A. Avoidable flaws in observational analyses: An application to statins and cancer. *Nat. Med.* **2019**, *25*, 1601–1606.
27. Dickerman, B.A.; García-Albéniz, X.; Logan, R.W.; Denaxas, S.; Hernán, M.A. Emulating a target trial in case-control designs: An application to statins and colorectal cancer. *Int. J. Epidemiol.* **2020**, *49*, 1637–1646.
30. Fu, E.L.; Evans, M.; Clase, C.M.; Tomlinson, L.A.; van Diepen, M.; Dekker, F.W.; Carrero, J.J.. Stopping Renin-Angiotensin System Inhibitors in Patients with Advanced CKD and Risk of Adverse Outcomes: A Nationwide Study. *J. Am. Soc. Nephrol.* **2021**, *32*, 424–435.
38. Kuehne, F.; Arvandi, M.; Hess, L.M.; Faries, D.E.; Matteucci Gothe, R.; Gothe, H.; Beyrer, J.; Zeimet, A.G.; Stojkov, I.; i Mühlberger, N.; et al. Causal analyses with target trial emulation for real-world evidence removed large self-inflicted biases: Systematic bias assessment of ovarian cancer treatment effectiveness. *J. Clin. Epidemiol.* **2022**, *152*, 269–280.
39. Kwee, S.A.; Wong, L.L.; Ludema, C.; Deng, C.K.; Taira, D.; Seto, T.; Landsittel, D.. Target Trial Emulation: A Design Tool for Cancer Clinical Trials. *JCO Clin. Cancer Inform.* **2023**, *7*, e2200140.
43. McGrath, L.J.; Nielson, C.; Saul, B.; Breskin, A.; Yu, Y.; Nicolaisen, S.K.; Kilpatrick, K.; Ghanima, W.; Christiansen, C.F.; Bahmanyar, S.; et al. Lessons Learned Using Real-World Data to Emulate Randomized Trials: A Case Study of Treatment Effectiveness for Newly Diagnosed Immune Thrombocytopenia. Clin. Pharmacol. Ther. 2021, 110, 1570–1578.
46. Schroeder, E.B.; Neugebauer, R.; Reynolds, K.; Schmittdiel, J.A.; Loes, L.; Dyer, W.; Pimental, N.; Desai, J.R.; Vazquez-Benitez, G.; Ho, P.M.; et al. Association of Cardiovascular Outcomes and Mortality With Sustained Long-Acting Insulin Only vs Long-Acting Plus Short-Acting Insulin Treatment. *JAMA Netw. Open* **2021**, *4*, e2126605.
47. Smith, L.H.; García-Albéniz, X.; Chan, J.M.; Zhao, S.; Cowan, J.E.; Broering, J.M.; Cooperberg, M.R.; Carroll, P.R.; Hernán, M.A. Emulation of a target trial with sustained treatment strategies: An application to prostate cancer using both inverse probability weighting and the g-formula. *Eur. J. Epidemiol.* **2022**, *37*, 1205–1213.
49. Talmor-Barkan, Y.; Yacovzada, N.S.; Rossman, H.; Witberg, G.; Kalka, I.; Kornowski, R.; Segal, E.; Head-to-head efficacy and safety of rivaroxaban, apixaban, and dabigatran in an observational nationwide targeted trial. Eur. Heart J. Cardiovasc. Pharmacother. 2022, 9, 26–37.

50. Trevisan, M.; Fu, E.L.; Xu, Y.; Savarese, G.; Dekker, F.W.; Lund, L.H.; Clase, C.M.; Sjölander, A.; Carrero, J.J. Stopping mineralocorticoid receptor antagonists after hyperkalaemia: Trial emulation in data from routine care. *Eur. J. Heart Fail.* **2021**, *23*, 1698–1707.
53. Xie, Y.; Bowe, B.; Gibson, A.K.; McGill, J.B.; Maddukuri, G.; Yan, Y.; Al-Aly, Z.Comparative Effectiveness of SGLT2 Inhibitors, GLP-1 Receptor Agonists, DPP-4 Inhibitors, and Sulfonylureas on Risk of Kidney Outcomes: Emulation of a Target Trial Using Health Care Databases. *Diabetes Care* **2020**, *43*, 2859–2869.
54. Xu, Y.; Fu, E.L.; Trevisan, M.; Jernberg, T.; Sjölander, A.; Clase, C.M.; Carrero, J.J. Stopping renin-angiotensin system inhibitors after hyperkalemia and risk of adverse outcomes. *Am. Heart J.* **2022**, *243*, 177–186.
55. Yarnell, C.J.; Angriman, F.; Ferreyro, B.L.; Liu, K.; De Grooth, H.J.; Burry, L.; Munshi, L.; Mehta, S.; Celi, L.; Elbers, P.; et al. Oxygenation thresholds for invasive ventilation in hypoxemic respiratory failure: A target trial emulation in two cohorts. *Crit. Care* **2023**, *27*, 67.
58. Zhang, Y.; Young, J.G.; Thamer, M.; Hernán, M.A. Comparing the Effectiveness of Dynamic Treatment Strategies Using Electronic Health Records: An Application of the Parametric g-Formula to Anemia Management Strategies. *Health Serv. Res.* **2018**, *53*, 1900–1918.
59. Admon, A.J.; Donnelly, J.P.; Casey, J.D.; Janz, D.R.; Russell, D.W.; Joffe, A.M.; Vonderhaar, D.J.; Dischert, K.M.; Stempek, S.B.; Dargin, J.M.; et al. Emulating a Novel Clinical Trial Using Existing Observational Data. Predicting Results of the PreVent Study. *Ann. Am. Thorac. Soc.* **2019**, *16*, 998–1007.
61. Cain, L.E.; Robins, J.M.; Lanoy, E.; Logan, R.; Costagliola, D.; Hernán, M.A. When to start treatment? A systematic approach to the comparison of dynamic regimes using observational data. *Int. J. Biostat.* **2010**, *6*, 18.
64. Kalia, S.; Saarela, O.; Escobar, M.; Moineddin, R.; Greiver, M. Estimation of marginal structural models under irregular visits and unmeasured confounder: Calibrated inverse probability weights. *BMC Med. Res. Methodol.* **2023**, *23*, 4.
65. Kuehne, F.; Jahn, B.; Conrads-Frank, A.; Bundo, M.; Arvandi, M.; Endel, F.; Popper, N.; Endel, G.; Urach, C.; Gyimesi, M.; et al. Guidance for a causal comparative effectiveness analysis emulating a target trial based on big real world evidence: When to start statin treatment. *J. Comp. Eff. Res.* **2019**, *8*, 1013–1025.
66. Lodi, S.; Phillips, A.; Lundgren, J.; Logan, R.; Sharma, S.; Cole, S.R.; Babiker, A.; Law, M.; Chu, H.; Byrne, D.; et al. Effect Estimates in Randomized Trials and Observational Studies: Comparing Apples With Apples. *Am. J. Epidemiol.* **2019**, *188*, 1569–1577.
